# Effects of resistance exercise in prostate cancer patients: A systematic review update as of March 2020

**DOI:** 10.1007/s00508-020-01713-x

**Published:** 2020-07-17

**Authors:** Andrej Zdravkovic, Timothy Hasenöhrl, Stefano Palma, Richard Crevenna

**Affiliations:** Department of Physical Medicine, Rehabilitation and Occupational Medicine, Medical University of Vienna, Waehringer Guertel 18–20, 1090 Vienna, Austria

**Keywords:** Resistance training, Prostatic neoplasms, Androgen antagonists, Sexual health, Systematic review

## Abstract

**Purpose:**

The aim of this systematic review is to provide an update on the effects of resistance exercise (RE) in patients with prostate cancer (PCa), with special attention to the effects on sexual health.

**Methods:**

A systematic search of the literature was conducted in March 2020 using the databases PubMed, MEDLINE, EMBASE, SCOPUS and the Cochrane Library. Only randomized, controlled trials published after 31 December 2016 were included in this update. Additionally, articles from current and previous reviews were utilized to provide a brief summary of the effects on sexual health.

**Results:**

A total of 10 articles met the inclusion criteria, of which 5 were identified as independent studies. The remaining five articles presented additional data for studies, which have already been included. The identified studies further strengthened the evidence for positive effects on muscle strength, body composition and physical function. Positive effects on bone mineral density were apparent only when RE was combined with impact training. One article reported an improvement in fatigue and health-related quality of life. Only one study examined the effects of RE in isolation and three articles indicated positive effects of exercise on sexual health.

**Conclusion:**

Recent evidence supports the use of RE in PCa patient rehabilitation as a countermeasure for treatment side effects. Further research is necessary to ascertain the optimal delivery methods and illuminate the effects on health-related quality of life (HRQOL), fatigue and sexual health.

## Introduction

Prostate cancer (PCa) is the most common malignancy in men [1] and shows the highest incidence rates of all cancer entities both in North America [2] and Austria [3]. It is responsible for the second most frequent cancer-related deaths in the USA [1, 2]; however, this is only because of the high absolute number of PCa patients, as the 5‑year and 10-year survival rates nowadays are well over 90% in developed countries [1, 4].

Although over 70% of men aged between 70 and 79 years will show histologic evidence of PCa, for the majority it is no longer the cause of death [1]. Therefore, for the majority of PCa patients their cancer has become a chronic, life-long, long-term disease. This increases the importance of supportive care in this patient population. A number of systematic reviews and meta-analyses have shown increases in quality of life [5], physical function and body composition [6] in PCa patients when conducting a resistance exercise (RE) program. From the perspective of exercise medicine RE is particularly beneficial for PCa patients as the side effects of their treatment often lead to a decrease in muscle mass and hence loss of physical function and increase of metabolic risk [7, 8] and RE is a potent intervention to counteract these complications [6].

Another major side effect of PCa treatment, particularly androgen deprivation therapy (ADT), is sexual dysfunction [9]. Sexual dysfunction finds its expression for the patients in decreased sexual desire, alone or together with erectile dysfunction, as well as absent orgasm [10]. The use of ADT is a major part of PCa treatment but alters men’s sexuality via loss of libido, the aforementioned erectile dysfunction, and testicular atrophy [11]. Treatment strategies for sexual dysfunction in PCa patients focus mainly on the treatment of erectile dysfunction after primary therapy; however, the sexuality issues of PCa patients are far more complex than erectile dysfunction alone and therefore require a multimodal treatment approach [12]. With its complex effects on various domains, such as physical fitness, quality of life, and perception of masculinity, RE has been proposed as a potentially effective treatment to maintain or even improve sexual health in PCa patients [13, 14].

Therefore, the primary aim of the current systematic review was to provide a systematic review update and qualitative overview about RE intervention trials in PCa patients published since 31 December 2016. The secondary aim was to draw particular attention to the outcome measures of parameters of sexual health in all RE intervention trials in PCa patients published to date.

## Patients, materials and methods

A systematic search of the scientific literature published from January 1966 until 31 March 2020 was conducted in the scientific databases PubMed, EMBASE, MEDLINE, SCOPUS and Cochrane Library using the search terms and Boolean operators ((resistance AND exercise) OR (resistance AND training) OR (strength AND training)) AND ((prostate AND cancer) OR (androgen AND (deprivation OR suppression) AND therapy)). Only randomized, controlled trials (RCT) examining the effects of RE in patients with PCa, published in English, were considered for this review. As this is an update to a previously published systematic review [5] and a meta-analysis [6], the main focus was on the recent studies published since 1 January 2017. The search strategy was congruent to those of the previous review papers of our research group [5, 6].

Search results were screened by title and abstract. Eligible articles underwent a full-text analysis. The literature search as well as the selection of suitable articles was performed independently by two experienced researchers. The individual evaluations of those researchers were brought together and, in cases of an unequal evaluation, discussed with inclusion of the senior author until a mutual evaluation was decided on. The number of patients per study arm, most relevant patient characteristics, details of the intervention and reported outcomes were extracted from each included independent study and the corresponding registered protocol. Furthermore, reported outcomes were extracted from the identified additional articles.

Risk of bias was assessed for the original studies published since 1 January 2017 utilizing the revised Cochrane risk of bias tool (RoB 2, current version as of 22 August 2019) at the level of the primary outcome for both the intention-to-treat effect and the per protocol effect.

In addition, included articles from the current and our previous reviews were searched and reports of effects on sexual health were extracted.

This review was conducted following the PRISMA reporting guidelines for systematic reviews and meta-analyses.

## Results

### Literature search

**Fig. 1:**
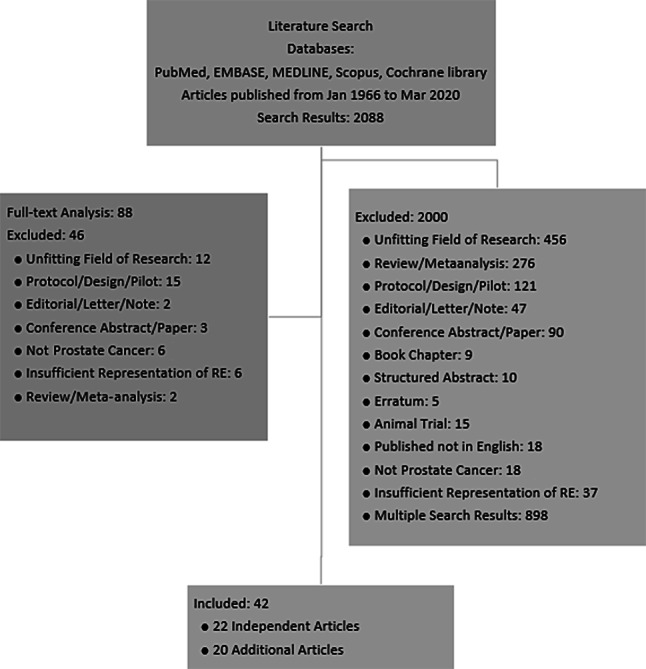
Flowchart of the systematic literature search and selection. *RE* resistance exercise

A total of 2088 articles were found and screened for eligibility by title and abstract. Of the articles 2000 did not meet the inclusion criteria, which left 88 articles for full-text analysis. Ultimately, 42 articles met the inclusion criteria of being relevant RE interventions with RE either as the sole exercise intervention or in combination with other exercise modalities in PCa patients [15–56]. Of those, 22 were identified as independent studies [15–31, 47–51], which published the primary outcomes in a specific population sample for the first time. In the other 20 articles, additional outcomes or analyses of former trials were published [32–46, 52–56]. Since 1 January 2017, 5 independent articles and 5 additional articles have been published. An overview of the selection process is shown in Fig. 1.

### Risk of bias

**Fig. 2:**
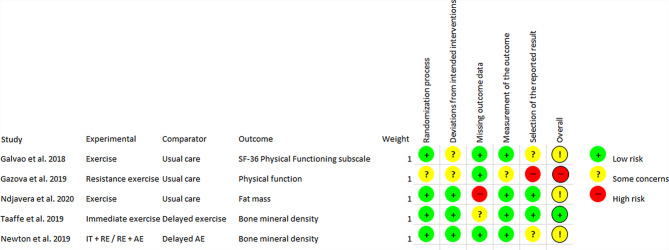
Risk of bias analysis, intention-to-treat aspect. *RE* resistance exercise, *AE* aerobic exercise, *IT* impact training

**Fig. 3:**
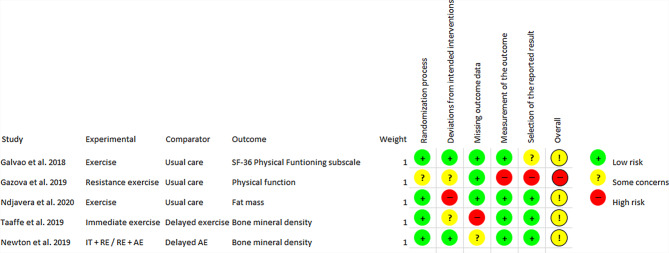
Risk of bias analysis, per protocol aspect. *RE* resistance exercise, *AE* aerobic exercise, *IT* impact training

The risk of bias analysis showed that the five independent articles published after 1 January 2017 were predominantly of a moderate to high methodological quality, with the exception of one study being classified as having a high risk of bias [47]. The results of the analysis for both the intention-to-treat effect are shown in Fig. 2 and for the per protocol effect in Fig. 3.

### Level of evidence

The level of evidence of the majority of the studies was graded as Ib, with the exception of one study, which was graded as IIb, due to low methodological quality. This study [47] suffered from a very small sample size, an insufficiently defined primary outcome, and within-group comparisons as the primary method of analysis. The level of evidence of the included studies is presented in Table 1.

**Table 1:** Level of evidence and type of exercise

Study	Year	Level of evidence	Type of exercise intervention
Gazova et al. [47]	2019	IIb	RE only
Galvao et al. [51]	2018	Ib	RE+AE+FLEX
Taaffe et al. [50]	2019	Ib	RE+AE+IT vs. delayed RE+AE+IT
Ndjavera et al. [48]	2020	Ib	RE+AE
Newton et al. [49]	2019	Ib	RE+IT and RE+AE vs. delayed AE

*RE* resistance exercise, *AE* aerobic exercise, *IT* impact training, *FLEX* flexibility training

### Independent articles

Apart from strengthening the evidence for the positive effects of RE on muscle strength, body composition, cardiovascular capacity, physical functioning and fatigue [47–50, 57], recent studies have indicated that a combination of RE and impact training (IT) decelerates the loss of bone mineral density (BMD) during ADT. Newton et al. [49] reported that a one year-long exercise intervention (RE + IT) resulted in a significantly lesser decrease in BMD, specifically at the lumbar spine and the femoral neck; however, the latter only at 6 months. Taaffe et al. [50] reported similar findings, although the between-group difference did not reach statistical significance. Finally, Gazova et al. [47] reported no effect of 4 months of RE on BMD. The results of the included studies are presented in Table 2.

**Table 2:** Study characteristics and main outcomes of the independent studies published since January 2017, details of previous studies published in Hasenoehrl et al. [5] and Keilani et al. [6]

Study	Sample	Mean age ± SD (years)	Patient details/cancer treatment	Duration of intervention (weeks)	Exercise program details	Frequency, duration and intensity	Key findings/comments
Newton et al. [49]	Impact training and RE (IRE) *n* = 57 RE and AE (RAE, Clinic-based for the first 26 weeks, home-based for further 26 weeks) *n* = 50 Delayed AE (DAE, usual care for the first 26 weeks, non-impact AE for further 26 weeks) *n* = 47	IRE 68.7 ± 9.3 RAE 69.1 ± 9.4 DAE 69.1 ± 8.4	Men treated for localized prostate cancer including ongoing ADT initiated at least two months before enrollment	52	RE: 2–4 sets of 6–12 repetitions (chest press, seated row, lat pull-down, leg press, leg extension, and leg curls). Bodyweight and resistance bands in the second half of the study for RAE AE: 20–30 min for RAE (various modalities), 30–40 min for DAE IT (hopping, leaping and drop jumping) FLEX (only DAE, modality not specified)	2 ×/week supervised exercise, 1 h/session IRE: 10 min warm-up RE at 12RM progressing to 6RM IT (mostly sets of 10 repetitions) 5 min cool-down Additional home-based IT 2 ×/week RAE: 10 min warm-up RE at 12RM progressing to 6RM AE at 70–90% of eHRmax 5 min cool-down Additional home-based AE to meet 150 min/week (same intensity) DAE: AE at approximately 70% of eHRmax	Primary outcome: Bone mineral density: Significantly lesser decrease for IRE in the lumbar spine at 26 and 52 weeks, femoral neck at 26 weeks Secondary outcomes: ↑ Muscle strength (IRE at 26 and 52 weeks, RAE at 26 weeks) ↑ Appendicular skeletal muscle mass (IRE at 26 weeks) ↔ Fat mass ↔ Lean mass Comparison with DAE
Taaffe et al. [50]	Immediate EI (IEI) *n* = 54 Delayed EI (DEI, usual care for the first 26 weeks) *n* = 50	Immediate EI 69.0 ± 6.3 Delayed EI 67.5 ± 7.7	Men treated for localized prostate cancer beginning ADT	26 Follow-up after 52	RE: 1–4 sets of 6–12 repetitions (leg press, leg extension, leg curl, chest press, seated row, lat pulldown and biceps curl) AE: 25–40 min (various modalities) IT (hopping, leaping and drop jumping)	3 ×/week supervised exercise, 1 h/session (3 × IT, 1–2 × RE, 1–2 × AE) 10 min warm-up RE varying between 6RM and 12RM or AE at 60–85% of eHRmax IT mostly sets of 10 repetitions 5 min cool-down Additional home-based AE and IT 2 ×/week	Primary outcome: bone mineral density: ↓ BMD for both groups over 52 weeks Tendency for lesser decrease for IEI after 26 weeks, DEI after 52 weeks Secondary outcomes: ↑ Fat mass (greater increase in DEI at 26 weeks) ↔ Lean tissue mass (IEI), ↓ in DEI at 26 weeks, ↑ at 52 weeks ↑ Self-reported physical activity (GodinQ) in IEI, (↑) in DEI ↑ Markers of bone turnover in IEI and DEI Substantially higher drop-out rate in DEI Some patients ceased ADT before the end of the study
Galvao et al. [51]	EI *n* = 28 Cont (usual care) *n* = 29	EI 69.7 ± 7.6 Cont 70.4 ± 9.3	Men with prostate cancer and bone metastatic disease	12	RE 3 sets of 10–12 repetitions (exercises targeting major muscle groups adapted according to site of metastatic disease) AE 20–30 min (various modalities) FLEX 2–4 repetitions of 30–60 sec (static stretching for all major joints)	3 ×/week supervised exercise, 1 h/session 10 min warm-up RE 10RM–12RM AE at 60–85% of eHRmax FLEX 5 min cool-down	Primary outcome: SF-36 physical function subscale: ↑ Physical functioning (EI) Secondary outcomes: ↑ Muscle strength (EI) ↔ Objective physical functioning (TUG, 6mWT, 400mWT) ↔ Body composition ↔ Fatigue (FACIT-F) No adverse effects of EI Patients with metastatic disease Missing values for several tests due to feasibility Complete case analysis
Ndjavera et al. [48]	EI *n* = 24 Cont (usual care) *n* = 26	EI 71.4 ± 5.4 Cont 72.5 ± 4.2	Men with prostate cancer beginning ADT	13, follow-up at 26	RE 2–4 sets of 10 repetitions (dumbbell squat, modified press-up, dumbbell bent-over row, dumbbell biceps curl, short arc quad, wall squat) AE 6 × 5 min with 2.5 min active recovery (aerobic interval training on a cycle ergometer)	2 ×/week supervised exercise, 1 h/session 5 min warm-up RE at 11–15 RPE (Borg 6–20) AE at 11–15 RPE (Borg 6–20) Advice to perform additional home-based AE and RE 3 ×/week	Primary outcome: Body fat mass: (↓) Body fat mass (EI) at 13 and 26 weeks Secondary outcomes: ↑ VO_2_max at 13 weeks, ↔ at 26 weeks (EI) ↓ Fatigue (FACIT-F) at 13 weeks, ↔ at 26 weeks (EI) ↔ Self-reported physical activity (GodinQ) at 13 weeks, ↑ at 26 weeks (EI) ↔ HRQOL (FACT-P) at 13 weeks, ↑ at 26 weeks (EI) ↔ Body composition ↔ Handgrip strength ↔ Metabolic profile
Gazova et al. [47]	EI *n* = 15 Cont (usual care) *n* = 8	EI 69.2 ± 5.8 Cont 70.7 ± 7.5	Men with localized prostate cancer on ADT	16	RE 2–3 sets of 10–15 repetitions (five exercises)	3 ×/week supervised exercise RE week 1–4 30% of 10–15RM, week 5–12 90–100% of 10–12RM, week 13–16 90–100% of 10–15RM	Primary outcomes: ↑Muscle strength (EI) ↔ Body weight ↔ BMI ↔ BMD ↑ 6MWT (EI) ↔ Stair climbing ↑ Metabolic profile (EI) Secondary outcome: ↑ Myogenic microRNA (EI) Small sample size High risk of bias Within-group comparison

*RE* resistance exercise, *AE* aerobic exercise, *IT* impact training, *FLEX* flexibility training, *EI* exercise intervention, *Cont* control, *ADT* androgen deprivation therapy, *eHRmax* estimated maximum heart rate (220 minus age), *BMD* bone mineral density, *GodinQ* Godin Leisure Time Questionnaire, *TUG* timed up and go test, *6mWT* 6m walk test, *400mWT* 400m walk test, *FACIT-F* Functional Assessment of Chronic Illness Therapy - fatigue, *HRQOL* health-related quality of life, *FACT-P* Functional Assessment of Cancer Therapy - Prostate Cancer, *BMI* body mass index, *6MWT* 6min walk test, ↑ significant increase or improvement, (↑) tendency for increase or improvement, ↓ significant decrease, (↓) tendency for decrease, ↔ no change

### Additional articles

Of the five additional articles, three presented similar findings in relation to muscle strength, cardiovascular capacity, fatigue body composition and physical functioning [52–54]. Edmunds et al. [56] provided a cost-effectiveness analysis of a 6-month intervention [21] and concluded that a clinic-based intervention should be expanded by a period of home-based exercise in order to achieve cost-effectiveness and that the inclusion of health-related quality of life (HRQOL) in the measurements might improve future economic evaluations. Fairman et al. [55] indicated that mere reporting of attendance might overestimate adherence to a RE protocol. The findings of the additional articles are presented in Table 3.

**Table 3:** Results of the additional articles published since January 2017, details of previous studies published in Hasenoehrl et al. [5] and Keilani et al. [6]

Additional article	Expansion	Key findings/comments
Taaffe et al. [53] Additional article of Newton et al. [49]	Additional results	↓ Fatigue (EORTC QLQ-C30 fatigue subscale, IRE at 26 and 52 weeks, RAE at 52 weeks, DAE at 52 weeks) ↑ Vitality (SF-36, IRE, RAE and DAE at 52 weeks) ↑ Cardiorespiratory fitness (400mWT, IRE and RAE at 52 weeks, no change in DAE) ↑ Muscle strength (sum of chest press and leg press, IRE at 26 < 52 weeks, RAE at 26 and 52 weeks, DAE at 52 weeks) No group × time interaction
Wall et al. [52] Additional article of Newton et al. [49]	Additional results RAE vs. DAE in the first 26 weeks	↑ Cardiorespiratory capacity (VO_2_max, RAE) ↑ Fat oxidation (RAE) ↔ RMR ↔ BP ↔ Arterial stiffness ↔ Metabolic profile ↔ PSA and testosterone ↑ Lean tissue mass (RAE) ↓ Fat mass (RAE) Between-group comparison
Fairman et al. [55] Additional article of Galvao et al. [51]	Additional results Training dose, adherence and tolerance	Actual training volume = 77.4% of prescribed Training interrupted (≥3 consecutive sessions missed) in half of patients Training missed (≤2 consecutive sessions missed) in approximately 90% of patients. Training dose modified in approximately 85% of patients
Edmunds et al. [56] Additional article of Galvao et al. [21]	Cost-effectiveness analysis	26 weeks of supervised exercise for prostate cancer survivors probably not cost-effective
Newton et al. [54] Additional article of Taaffe et al. [50]	Additional results	↑ Muscle strength – In IEI at 26 weeks – In DEI at 52 weeks – No difference at 52 weeks ↑ Physical function – 6mWT (only in IEI at 26 weeks) – 400mWT (IEI at 26 weeks, DEI at 52 weeks) – Stair climbing (IEI at 26 weeks, DEI at 52 weeks) – Repeated chair rise (IEI at 26 weeks, DEI at 52 weeks) – Significant difference only for 6mWT at 52 weeks Between-group comparison

*IRE* impact training and resistance exercise group, *RAE* resistance and aerobic exercise group, *DAE* delayed aerobic exercise group, *VO*_*2*_*max* maximal oxygen uptake, *RMR* resting metabolic rate, *BP* blood pressure, *PSA* prostate-specific antigen, *IEI* immediate exercise intervention group, *DEI* delayed exercise intervention group, *6mWT* 6m walk test, *400mWT* 400m walk test, ↑ significant increase or improvement, ↓ significant decrease, ↔ no change, *EORTC QLQ-C30* European Organization for Research and Treatment of Cancer Quality of Life Questionnaire

### Sexual health

When looking at the outcomes regarding sexual health in all included studies, sexual health was reported as a secondary outcome in only three articles [18, 34, 45]. The instrument used in two of these [18, 34] was a disease-specific questionnaire (EORTC QLQ-PR25 – European Organization for Research and Treatment of Cancer Quality of Life Questionnaire—Prostate), whereas in the third [45], a modified questionnaire according to Druley et al. [58] was completed by PCa patients and their spouses. No objective measurements of sexual dysfunction were performed. Self-reported sexual function [18] as well as sexual activity and interest in sex [34] significantly improved in the exercise intervention group. Partnered exercise led to an increased frequency of engagement in affectionate behavior in partners but not in PCa patients themselves. The results regarding sexual health are outlined in Table 4.

**Table 4:** Results of studies assessing the effects of resistance exercise on sexual health in prostate cancer patients

Study	Sexual health-related outcome	Results
Lyons et al. [45] Additional article of Winters-Stone et al. [30]	Levels of physical intimacy	Engagement in affectionate and sexual behavior – Engagement in affectionate behavior: ↑ in spouses, ↔ in patients – Engagement in sexual behavior: ↔ in patients and spouses
Cormie et al. [18]	Disease-specific health-related quality of life (EORTC QLQ-PR25)	↑ Physical, mental and sexual function
Cormie et al. [34] Additional article of Galvao et al. [20]	Disease-specific health-related quality of life (EORTC QLQ-PR25)	Comparison EI vs. Cont postintervention: ↑ Sexual activity in EI ↑ Major interest in sex in EI (↑) Any level of interest in sex in EI Significant associations: – Change in sexual activity postintervention with change in perceived general health and role-emotional

EI exercise intervention, Cont control, ↑ significant increase, (↑) tendency for increase, ↔ no change

## Discussion

Since January 2017, 10 articles have been published on the subject of RE in PCa patients: 5 independent studies and 3 additional articles expanded the evidence for the positive effects of RE on muscle strength, body composition, cardiovascular capacity, physical functioning and fatigue [47–50, 52–54, 57]. Of the independent studies two also indicated that a combination of RE and IT decelerated the loss of BMD during ADT.

Taaffe et al. [50] investigated the effects of an exercise intervention beginning approximately at the same time as ADT or 6 months later. The intervention, consisting of RE, aerobic exercise IT and stretching attenuated the loss of muscle mass and gain in fat mass. No effect on BMD was seen at 12 months, as it decreased in both groups; however, the partial cross-over design of the trial may have diminished the group differences. Even though the delayed intervention failed to attenuate the loss of BMD, it may be prudent to recommend an immediate initiation of exercise in this patient population, as bone metabolism is different and the recovery of BMD may be slower in older age [59].

The study by Galvao et al. [57] was the only one conducted in patients with metastatic disease. The exercise protocol, lasting 3 months and consisting of RE, aerobic exercise and stretching, was adapted for each patient, according to site of bone lesions. Due to difficulties in performing the majority of the tests, only complete cases were analyzed. The exercise intervention resulted in significant increases in muscular strength and self-reported physical functioning. Most probably, the clinically most important finding was the overall good acceptance and tolerance of the RE intervention. For this particular patient population, interventions affecting the risk of fractures and falls are of great interest. In a recent international cohort study on community-dwelling older men, the risk of a major osteoporotic fracture was negatively correlated with walking speed, grip strength and appendicular lean mass [60]. Furthermore, a recent systematic review found that interventions implementing RE, coupled with balance and functional exercises, probably reduced the risk of falls in older adults [61]. Whether these findings can be extrapolated to a reduced risk of fractures and falls in PCa patients with bone metastases remains to be determined, but there is currently little evidence to suggest otherwise.

Ndjavera et al. [48] implemented a 3-month exercise intervention of RE and interval aerobic exercise at the start of ADT, with a follow-up at 6 months. Improvements in fat mass, cardiovascular capacity, self-reported physical activity and HRQOL were observed, which further support the notion of concurrent ADT and exercise, similarly to the findings of Taaffe et al. [50].

Newton et al. [49] reported the results of a three-armed, one year-long RCT on PCa patients during ADT. In the study RE was combined either with aerobic exercise or IT and compared with a delayed intervention group. Bone loss was significantly slower in the RE + IT group at 6 months, but statistical significance was lost at 1 year for most sites. Interestingly, appendicular skeletal muscle mass improved only in the RE + IT group, even though both RE regimens resulted in an increase in muscle strength. The possibility of interference of RE and aerobic exercise, as well as additional loading due to IT were discussed by the authors.

Gazova et al. [47] presented the findings of a small RCT examining the effects of 16 weeks of isolated RE in PCa patients on ADT. The authors noted an improvement in muscle strength, cardiovascular capacity and, as a secondary outcome, an increase in the plasma concentration of myogenic miRNA. The study exhibited several potential methodological limitations. The sample size was small, comprising 15 patients in the intervention group and 8 in the control group, which may have increased the probability of a type II error [62]. Moreover, no sample size calculation was reported, which limits the ability to estimate the statistical power of the depicted results. The RE intervention was not sufficiently defined according to the FITT formula (Frequency, Intensity, Time and Type of exercise), which in turn reduces the detailed estimation of the RE stimulus as well as the reproducibility of the trial [63]. A total of 19 primary outcome measures were reported, alongside 3 secondary outcome measures. According to the CONSORT statement [64], a large number of primary outcomes increases the probability of a type I error; however, whether a type I or type II error truly occurred, cannot be determined at this time point [62]. Nevertheless, contrary to what one would expect of an RCT, the primary method of analysis used were within-group comparisons instead of between-group comparisons. This defeats the purpose of an RCT and increases the probability of a type I error [65].

Regarding the results of the additional articles, reported secondary outcomes indicated improvements in muscle strength [53, 54], cardiovascular capacity [52–54], body composition [52], fatigue [52] and physical functioning [54]: however, the majority of the between-group comparisons showed no statistically significant differences. The reason for this might be that sample size calculations are always based on the primary outcome parameter which is normally published in the original article of a larger study. The secondary outcome parameters of additional articles can therefore never be treated with the same emphasis. These non-significant results are partly in line with the findings of our previous systematic review and meta-analysis [5, 6]. There, significant improvements in body composition, e.g. lean body mass and body fat, and in the 400m walk time could be found in the pooled results although the individual results of the vast majority of the pooled studies did not show significant results [6]. Pooling and therefore meta-analysis regarding fatigue and HRQOL was, and still is, impossible due to the high variability of outcome measures of these outcomes [66]; however, as RE is known to reduce cancer-related fatigue in general [67] as well as physical functioning in older adults [68], it is most probably a matter of time and not of validity before specific recommendations, concerning the implementation of RE to counteract fatigue in PCa patients can be made.

Sexuality and sexual health are an important aspect of life for a large proportion of people, even in old age, although to a lesser degree [69, 70]. Sexual bother, which is defined as distress caused by sexual dysfunction, and erectile dysfunction are highly prevalent in PCa patients, especially when the treatment includes ADT [13]. Furthermore, erectile dysfunction has been linked to depression in this patient population [71]. It is important to note that sexual bother is not purely a result of erectile dysfunction, as it can persist even after the return of erectile function [72]. Other factors negatively influencing sexual health include body feminization, fatigue, depression and a reduced HRQOL [73]. Moreover, the fat-free mass index has in itself been associated with sexual desire in middle-aged men [74]. Beside pharmacological interventions, exercise [13] and psychological counselling, the latter including education on alternative sexual practices [75], have been suggested as complementary therapeutic approaches. Despite the high prevalence and subjective disease burden of sexual dysfunction and the ever increasing commonness of RE interventions during ADT, only three studies [18, 34, 45] investigated the effects of RE on sexual function. Utilizing the EORTC QLQ-PR25, Cormie et al. [34] reported a significant increase in self-reported sexual activity and interest in sex following a 3-month RE and aerobic exercise intervention. Interestingly, the change in sexual activity correlated to perceived general health and role-emotional. The same research group reported the results of a second trial 1 year later [18], indicating positive effects of a similar exercise intervention on the HRQOL and in particular the self-reported sexual function, as measured by the same questionnaire. Lyons et al. [45] however, employed a different approach and examined the effects of a partnered RE intervention on self-reported physical intimacy in PCa patients and their spouses. The participants answered questions regarding affectionate, as well as sexual behavior on a 4-level Likert scale, as modified from Druley et al. [58]. Curiously, only the females reported an increase in affectionate behavior, and neither reported an increase in sexual behavior. Nevertheless, the results of these studies indicated positive effects on sexual health in PCa patients, and one drew the conclusion that future research should put more emphasis on this field. As it is understandable that side-effects of PCa therapy, such as fatigue, loss of muscle mass and pelvic floor disorders influence sexual health, it is possible that many of these symptoms persist due to a vicious cycle of weakness, fatigue and depression, being that greater muscle strength has been associated with lower fatigue in cancer survivors [76]. In this respect, RE, primarily with its effects on muscle mass and strength, including that of the pelvic floor [77], may provide an efficient therapeutic approach for sexual dysfunction in PCa patients. The optimal timing, dose and modality should be the subject of future research.

However, recommendations regarding physical activity are not always met in the general population, in particular as pertaining to RE, and this omission has been associated with greater deficits in the activities of daily living [78]. Although certain idiosyncrasies of cancer patients, especially those with bone metastatic disease, have to be considered [79], public awareness of the positive effects of exercise, including the socioeconomic impact, should be raised [80] in order to facilitate the application of the findings of clinical studies.

The limitations of this systematic review update are primarily twofold. Firstly, half of the articles published since 1 January 2017 presented additional data of independent articles. As mentioned before, the outcome parameters of additional articles cannot usually be graded with the same statistical weight as the original papers whose sample size had been calculated for the primary outcome measure in the first place and not for the additional outcomes. Therefore, the results of those articles generally must be treated with caution. Secondly, due to the sparse literature and heterogeneous assessment methods no meta-analysis of parameters of sexual health was possible. Future research should work toward closing this knowledge gap and thus enabling a meta-analysis of the cumulative data and formulation of concrete recommendations.

## Conclusion

The results of this systematic review update expand the evidence for the efficacy of RE in the rehabilitation of PCa patients. Recent data also indicated that a combination of RE and IT has positive effects on BMD during ADT. Only a few studies have investigated the effects of RE on sexual health in this patient population. Considering their promising results, RE as a strategy to approach sexual health-related side effects in PCa patients is inadequately represented in the current literature and deserves a larger focus in future research.
